# Sex differences in asbestos exposure

**DOI:** 10.3389/fpubh.2025.1588415

**Published:** 2025-05-22

**Authors:** Krishna Patel, Stephanie Tuminello, Emanuela Taioli

**Affiliations:** Department of Thoracic Surgery, Icahn School of Medicine at Mount Sinai, Institute for Translational Epidemiology, New York, NY, United States

**Keywords:** asbestos exposure, cancer risk, environmental risk, non-occupational exposure, epidemiology

## Abstract

**Background:**

Although the association between exposure to asbestos and malignant mesothelioma has been established, occupational exposure has been historically present in males, while the ascertainment of female exposures is more nuanced. We reviewed the literature to assess differences in environmental exposure in mesothelioma cases according to sex.

**Methods:**

A new PubMed search was conducted with the key words “mesothelioma” and “environmental exposure” on October 11, 2024 with a start date of January 1, 2016, to supplement our previous qualitative review that included publications up through June 2016. Studies conducted in occupational settings were excluded.

**Results:**

Out of the 26 eligible papers, 11 were excluded because they did not report information on exposure by sex, leaving 15 published studies that were added to the 9 from our previous qualitative synthesis (24 total studies). 19 studies were cross-sectional, 2 were cohort and 3 were case control studies. The average NIH Study Quality tool score was 7.4/14 (minimum 3, maximum 12). Occupational exposure was more frequently observed in males than in females. While a male to female ratio favored males, there was variation in the strength of the association. There was a large proportion of cases with “unknown exposure,” and these were more frequently observed among female cases. In some studies, up to 40% of female cases had unknown exposure profiles. Quality assessment showed a generalized lack of standardization in the definition of environmental exposures across studies.

**Conclusion:**

Although recent studies have continued to improve our understanding of environmental exposure to asbestos and other elongated fibers, challenges remain, including but not limited to lack of rigorous, high-quality evidence and difficulty standardizing definitions across countries and datasets to enable appropriate comparison across studies.

## Introduction

Although the association between exposure to asbestos and malignant mesothelioma has been established, the bans and restrictions on asbestos use, production and import have contributed little to curb the incidence of mesothelioma in the US and around the world in the population at large (1). Some of the reasons for this discrepancy between the implementation of stricter regulations and the observed flat curve of mesothelioma incidence may be explained by both the long latency period of the disease, which usually presents 30 to 40 years after exposure. Moreover, asbestos fibers have persisted and remained in the environment, even after the new regulations (2). Males have historically experienced occupational asbestos exposure, and while this exposure has been reduced by bans on production and use, asbestos is still present in several structures including schools, municipal buildings, and in residential areas built in close proximity to former asbestos mines, factories, and soil containing natural asbestos.

As such, it is more difficult to ascertain and quantify these forms of non-occupational exposures, which are thus understudied (3). Non-occupational asbestos exposure may affect females more often than males and may go unnoticed unless specific questions and screening are carried out in a systematic manner. Trends in U.S. mesothelioma incidence suggest that incidence among males has decreased in recent years, while female incidence rates have been stable (4). This data suggests that there may be gender disparities in terms of asbestos exposure type, length of exposure, and at-risk populations, in addition to temporal changes in other risk factors such as smoking behavior. Here, we review the literature on mesothelioma in non-occupational settings by sex to explore these disparities and offer possible future directions in mesothelioma prevention.

## Methods

We previously published a comprehensive qualitative review of studies on environmental exposure and mesothelioma that included publications up to June 2016 (5). To supplement this, we updated the published manuscript by conducting a new PubMed search with the key words *mesothelioma* and *environmental exposure* on October 11, 2024 with a start date of January 1, 2016 (5). Description of asbestos exposure according to sex was also added as an additional inclusion criterium.

The flowchart of study selection and review is outlined in Supplementary Figure 1. From 1/1/2016 to 9/30/2024, there were 645 studies published in English. After reviewing the abstracts, we further excluded articles that were not pertinent to the study question (*n* = 516). The remaining 129 papers were reviewed, with 103 papers further excluded because they were reviews, case reports and comments, editorials, or letters. This left 26 eligible papers whose full text was assessed; 11 studies were excluded because they did not report information on asbestos exposure by sex. This left 15 studies reporting exposure data stratified by sex that were added to the 9 publications in our previous qualitative synthesis. The overall quality of the 24 papers was assessed by two investigators (K.P. and E.T.) who separately addressed 14 points from the quality assessment tool developed by National Institutes of Health for observational cohort and cross-sectional studies. The 14 points in the quality assessment tool are weighted equally, with a maximum possibility quality score of 14, and encompass criteria of methodology rigor including but not limited to clarity of research question and study population, measurements of outcomes and exposure, and blinding of outcome assessors (6).

In cases of disagreement, the two investigators convened and discussed the reasons behind their score to reach a consensus. The summary quality scores are reported in Table 1. Despite anticipation of high heterogeneity among the reviewed studies in their design and in their measures for exposure and health outcomes, a quantitative summary estimate was conducted.

**Table 1:** Description of the studies included in the review.

Reference	Location	Data source/instrument for exposure assessment (study period)	Exposure type	Study type	N (females)	Asbestos type	Quality score
Italy							
(7)	Veneto region	National Mesothelioma Registry (1987–2010)	O and EE	Cross-sectional	1,600 (422)		8
(8, 9)	Italy	National Mesothelioma Registry (1993–2008)	O and EE	Cross-sectional	15,322 (4,358)		8
(10–12)	Italy	ReNaM registry, 1993–2012	O, EE, NOA	Cross-sectional	19,955 (5496)		6
(13)	Friuli VG, Italy	Friuli mesothelioma registry 1995–2014	household	Cross-sectional	35 (2)	shipyard	5
(15–17)	Lombardy, Piedmont region	National Mesothelioma Registry (2000–2016)	O and EE	Cross-sectional	4,442 (1,592)	Chrysotile, crocidolite, amosite	8
(18)	Lombardy, Italy	Lombardy mesothelioma Registry 2000–2016	O, EE, NOA	Cross-sectional	6,226 (2,178)	Amphiboles, Chrysotile	9
(29)	Lombardy, Italy	Lombardy mesothelioma Registry 2000–2016	O, EE, NOA	Cross-sectional	218 (121)		8
(19)	Lombardy, Italy	Lombardy mesothelioma Registry 2000–2019	O, EE, NOA	Cross-sectional	562 (190)	Amphiboles, Chrysotile	8
(14)	Emilia Romagna, italy	ER mesothelioma registry, 1996–2021	O, EE, NOA	Cross-sectional	2,683 (704)	Amphiboles, Chrysotile	6
(26)	Bari, Italy	Apulia Meso registry, 1989–2019	EE	Cross-sectional	71 (40)	Amphiboles, Chrysotile	10
Australia							
(30)	West Australia	Western Australian Mesothelioma Register (1960–2008)	O and EE	Cross-sectional	1,631 (223)	Crocidolite	8
Other EU							
(31)	France	National Mesothelioma Surveillance Program (1998–2008)	O and EE	Cross-sectional	1,937 (411)		8
(25)	France	National Mesothelioma Surveillance Program (1998–2002)	O, non O	Case–control	437 (75)		8
(20)	Denmark	Pathology cases 1974–2015, females	O, EE, NOA	Cross-sectional	91 (91)	Chrysotile, amosite, crocidolite	9
(21)	Denmark	7^th^ grade School cohort born 1940 to 1970	EE	cohort	6 (6)	80% chrysotile	10
(22)	Denmark	Hospital registry 1996–2012	O, EE	Cross-sectional	30 (30)		5
(39)	Metsovo, Greece	local hospital, municipality mortality data (1995–2009)	NOA, Others	Cross-sectional	12 (9)	tremolite, erionite	5.5
USA							
(32)	Minnesota, US	1988–2010	O, non O	cohort	4 (3)	Libby vermiculite	12
(27)	US various	Lawsuit	EE (talc)	Case series	75 (64)	Anthophyllite, tremolite	4
(28)	US various	Lawsuit, 2014–21	EE (talc)	Case series	109 (83)	Anthophyllite, tremolite	3
Others							
(40)	South Africa	Asbestos Relief Trust compensation database (2003–2010)	EE	Cross-sectional	77 (34)	Crocidolite, amosite, chrysotile	5
(24)	Sibate’, Colombia	Health survey 2015	O, EE, NOA	Cross-sectional	13 (4)	Crocidolite, chrysotile	6
(23)	Amagasaki city, Japan	1975–2002	O, EE, NOA	Case–control nested	133 (49)	Crocidolite, chrysotile	
(41)	Sivas, Turkey	Residents	EE	Case–control	100 (45)	ophiolites	8

O = occupational exposure; EE = environmental exposure; NOA = naturally occurring asbestos.

## Results

Of the 24 papers covering 29 datasets included in this review, 19 studies were cross-sectional, 2 were cohort, and 3 were case control studies (Table 1). The average NIH Study Quality tool score was 7.4/14 (minimum 3, maximum 12). Assessments of exposures were captured as part of this quality tool, and both male and female patients were assessed in the same way through national registries built from standardized questionnaires and survey data or direct interviews of patients. Studies were conducted in Italy, Australia, Denmark, France, Greece, various locations in the US, South Africa, Colombia, Japan, and Turkey. Manuscripts reporting on the same or overlapping sample of cases were considered together, and only the most updated version of the results was reported in Table 1 and analyzed subsequently.

### Definitions of exposure

A summary of the different definitions of non-occupational exposure used by the included studies is reported in Table 2. Eight studies from the Italian registry (7–14) classified non-occupational exposure into familial, environmental and “hobby related.” Another group of investigators (15–19) used categories of “familial” or “para-occupational” relating to domestic exposure (repairs, ironing etc), as well as “environmental indoor” (material in the house) and outdoor (living near a factory). Some investigators (20–23) further defined domestic exposure or household exposure (24) as living with a person with occupational exposure, while other investigators (25) defined domestic exposure as any of having a family member occupationally exposed, having asbestos devices at home, or involvement in do-it-yourself home projects.

**Table 2:** Definitions of non-occupational exposure.

Reference	Definition
(7–14)	familial, environmental and hobby
(15–19)	familial (or para-occupational, clothes), domestic (repairs, ironing), environmental indoor (material in the house) outdoor (living near a factory)
(29)	familial, environmental (living close to)
(26)	familial or para-occupational, domestic or home-related, environmental, leisure
(30)	Residence, home renovators, other
(31)	Domestic exposure: family member, asbestos devices at home, DIY
(25)	Domestic (DIY), para-occupational (clothes) and environmental (living close to)
(20)	Domestic (living with), environmental (living close to)
(21)	Domestic (living with), environmental (school)
(22)	Domestic (living with)
(32, 39–41)	ambient (residence)
(27, 28)	Cosmetic talc
(24)	Environmental (residence), household (living with), non-professional
(23)	Domestic (living with), household (material in the house), residence (living close to)

### Characteristics of cases according to type of exposure

Of the 24 included studies, 11 reported data for pleural mesothelioma (7, 12, 13, 15, 16, 19, 22, 24–28), and the other 13 reported on mesothelioma in general without a distinction between pleural and other sites.

### Male vs. female type of exposure

Six studies reported on past occupational exposure by sex in patients with pleural mesothelioma (7, 12, 15, 16, 19, 24, 25), while eight studies reported this information on mesothelioma in general without distinction by site (8, 9, 14, 18, 23, 29–32).

**Figure 1:**
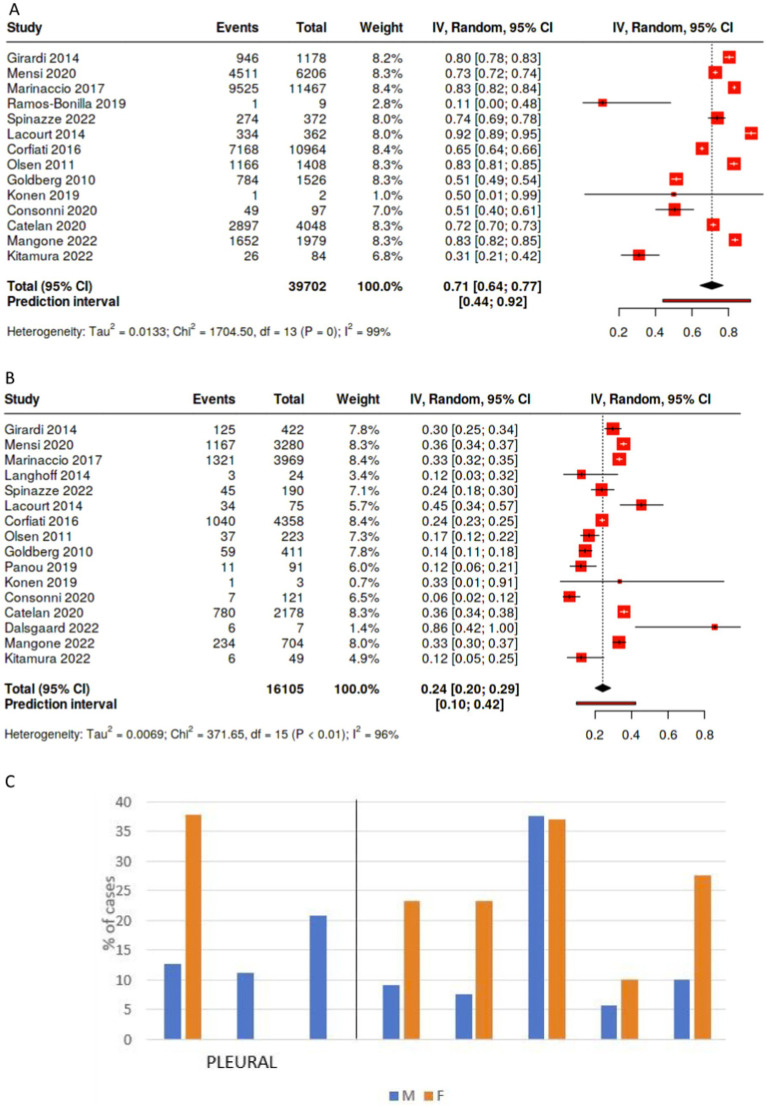
Percentage of cases with recorded occupational exposure to asbestos by sex*. **(A)** Percentage of male patients with recorded occupational exposure to asbestos. **(B)** Percentage of female patients with recorded occupational exposure to asbestos. *17/24 studies are shown that reported data on male and female past occupational exposure. **(C)** Male to female ratio, occupational exposure.

**Figure 2:**
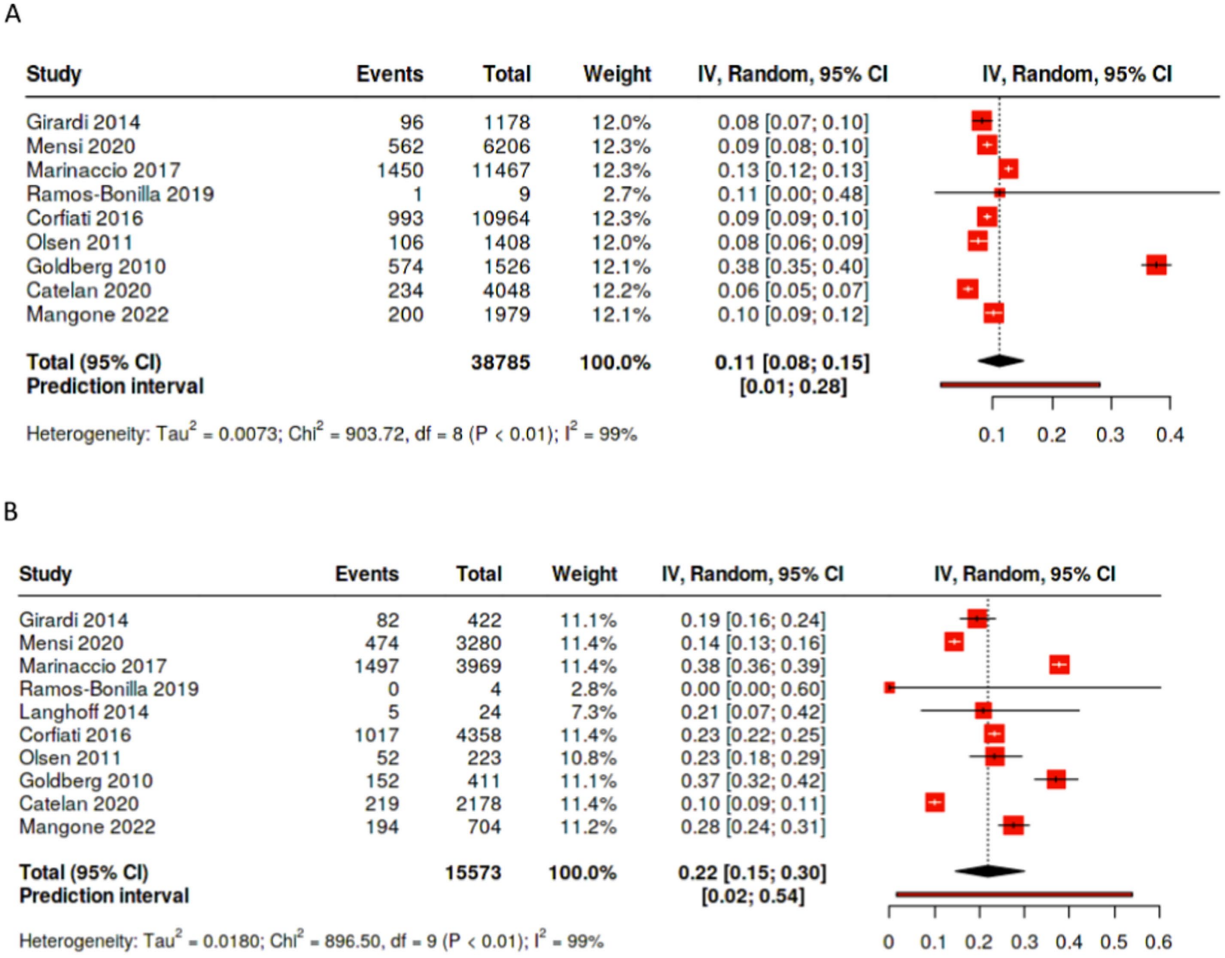
Percentage of cases with unknown exposure by sex. **(A)** Percentage of male patients with unknown exposure to asbestos. **(B)** Percentage of female patients with unknown exposure to asbestos.

**Figure 3:**
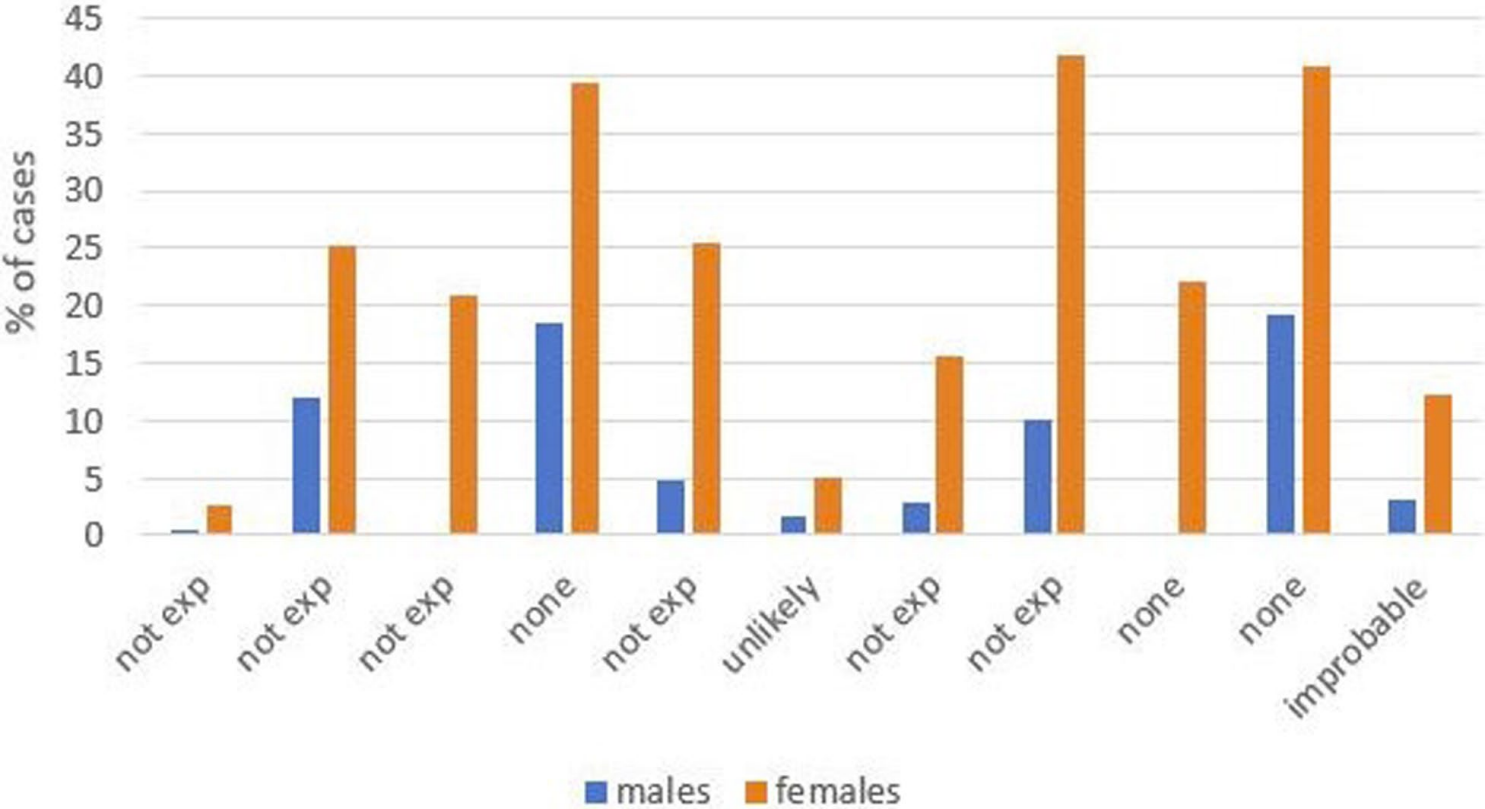
Proportion of cases with no history of exposure by sex.

Occupational exposure was more frequently observed in males than in females; among studies reporting on past occupational exposure by sex, the male to female ratio favors males, although the strength of the association varies (Figures 1A,B). In studies including both sexes with more than 30 cases, the percentages of occupational exposure cases consisting of men ranged from 78.7% (18) to 96.9% (30). Moreover, not only are there a large number of cases with unknown exposure, the proportion of unknown exposure cases was seen relatively more frequently among female patients (Figures 2A,B, 3). In the majority of studies, females had greater proportions of cases with unknown exposure compared to males, with some studies reporting as high as 25% of female cases having unknown exposure (Figure 3B). Across all studies of female patient cohorts with multiple sources of exposure, the percentages of mesothelioma cases attributable to unknown exposures ranged from 9% (12) to 22% (19).

**Figure 4:**
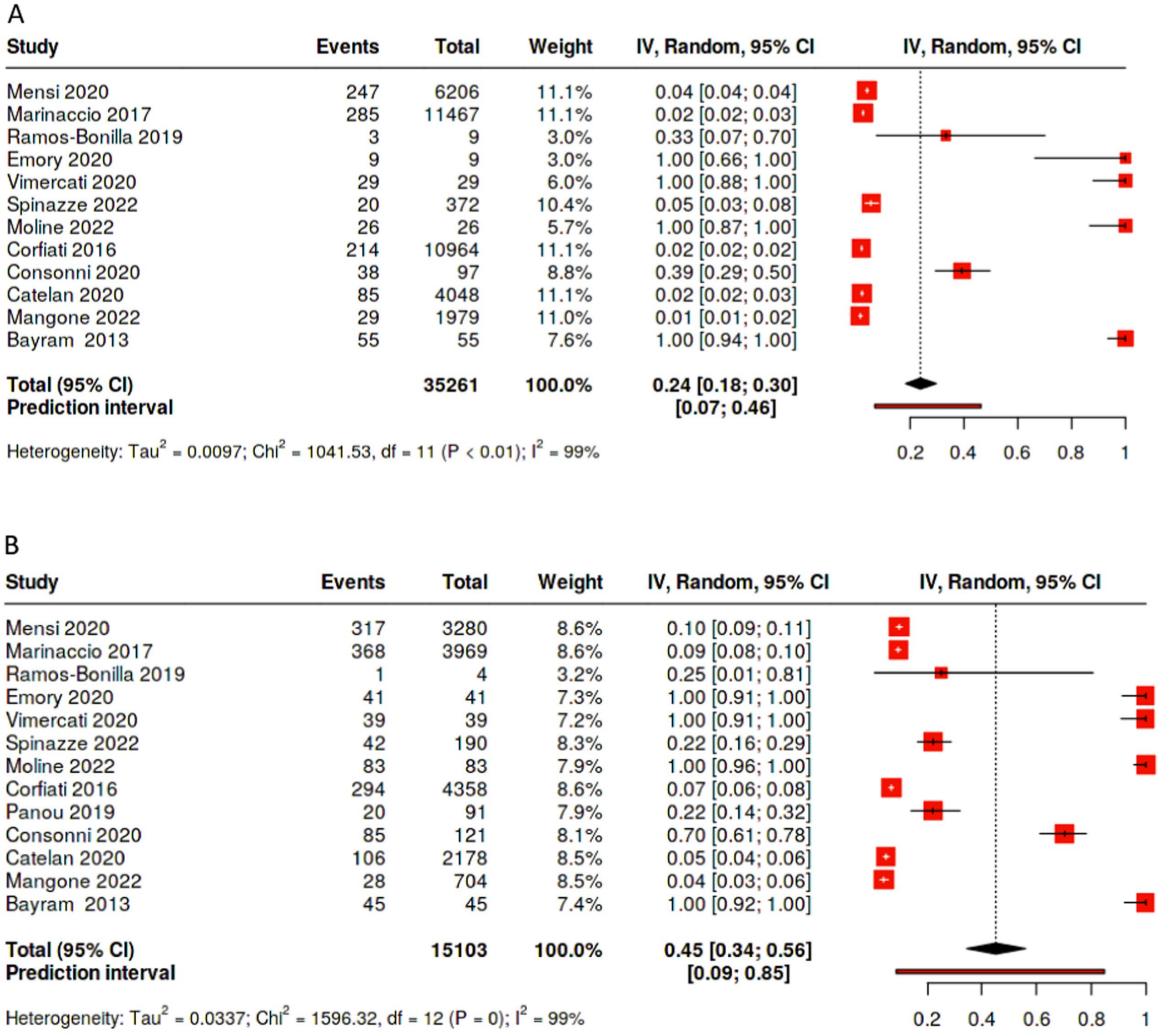
Percentage of cases with environmental exposure to asbestos by sex. **(A)** Percentage of male patients with unknown exposure to asbestos. **(B)** Percentage of female patients with unknown exposure to asbestos.

Overall, pooling data across studies showed that the weighted pooled proportion of cases with environmental exposure was significantly higher among female (0.45 [95% CI: 0.34, 0.56], *p* < 0.01, *n* = 15,103) than male mesothelioma cases (0.24 [95% CI: 0.18, 0.30], *p* < 0.01, *n* = 35,261; Figures 4A,B). Likewise, the proportion of females mesothelioma linked with unknown exposure (0.22 [95% CI: 0.15, 0.30], *p* < 0.01, *n* = 15,573) is significantly greater the proportion of males (0.11 [95% CI: 0.08, 0.15,], *p* < 0.01, *n* = 38,785; Figures 2A,B), with large statistical heterogeneity. The pooled and weighted proportion of occupational exposure among male mesothelioma cases was 0.71 (95% CI: 0.64, 0.77, *p* < 0.01; *n* = 39, 702) significantly higher than that observed among female cases at 0.24 (95% CI: 0.20, 0.29; *p* < 0.01; *n* = 16,105; Figures 1A–C) with large statistical heterogeneity.

## Discussion

This review of publications on mesothelioma cases from non-occupational settings provides evidence suggesting gender disparities in asbestos exposure modalities. Among cases reporting a past occupational exposure, males are by far the predominant sex, both for pleural mesothelioma and mesothelioma in general, as shown by uniform positive male to female ratios across studies. When other forms of non-occupational exposures are analyzed, females are more likely to report unknown or no exposure at all, confirming the need to more extensively query these cases about their past asbestos exposure history. It is possible that local residents may be less aware of the presence of environmental asbestos exposure, thereby reducing the likelihood of non-occupational exposures being reported when asked. The disparities by sex data suggest that there are sources of asbestos exposure that are not apparent or known to the cases. Therefore, without the necessary awareness, these individuals are more likely to have made no attempt to avoid such exposure and are therefore at increased risk of the disease. In recent years, other uncommon sources of asbestos contamination have surfaced, including cosmetic products and household items such as home decorations and textiles (27, 28). These products’ composition may be prone to contamination and content are often not appropriately labeled, and further contribute to under-reporting of exposure and lack of awareness by patients.

From a public health perspective, updating exposure questionnaires to include novel, uncommon sources of exposure, along with standardized methods to collect exposure information are needed. Mineralogical studies to identify geographic areas with natural occurring asbestos-like fibers are also needed, so that a map of exposure can be created and assessed in relation to residential history of cases, without relying on individual patient recall or knowledge of their residential exposure. Appropriate linkage between administrative health data, such as cancer registry and SPARCS, and mineralogical datasets and other useful exposure datasets provided by the U.S. Environmental Protection Agency such as the National Air Toxics Assessment (NATA) data, could help identify risk from inhalation of non-asbestos air toxins, emission sources, meteorological conditions, human activity patterns, smoking, and iatrogenic causes including radiation therapy. Finally, appropriate residential history should be coupled with emerging geospatial data-collection methods to avoid recall bias and bolster internal validity (33).

More precise measurements of asbestos exposure are urgently needed to better characterize risk, given the high proportion of cases where a clear exposure cannot be identified through questionnaires or other environmental measures. For instance, while certain biomarkers like the detection of asbestos fibers in mesothelioma tissue can be a proxy of past exposure, they are still imperfect measures of asbestos exposure and would therefore not be helpful for preventive purposes and predicting risk (34). Most of the existing biomarkers are geared toward early detection of mesothelioma rather than asbestos exposure (35). Mesothelin and osteopontin have showed early promise for early detection of asbestos-related mesothelioma; when measuring soluble mesothelin-related peptide (SMRP) levels in malignant pleural mesothelioma as well as benign pleural pathology, SMRP was able to distinguish between asbestos exposed and naïve patients (36). The prognostic capacity of osteopontin for detecting malignant mesothelioma was evaluated in a six-study meta-analysis and showed an overall specificity, sensitivity, and area under curve were 81, 65%, and 0.83, respectively. Moreover, the diagnostic odds ratio of osteopontin was 10.65 (95% CI: 7.13–15.91), and the positive and negative likelihood ratios were 3.77 (95% CI: 1.82–7.82) and 0.42 (95% CI: 0.31–0.58), respectively (37). However, none of these biomarkers can distinguish patients at risk for mesothelioma while they are still healthy.

Proteomic and epigenomic studies have also shown some promise in identifying asbestos-related profiles, but the translational potential is still in its early stages. Generic biomarkers like oxidative stress, inflammation, gene expression have not offered the specificity necessary for detection of asbestos exposure in healthy subjects (34). For instance, while one study found mir-29c mi-RNA was associated with worse prognosis independent of malignant pleural mesothelioma histology (*n* = 75), there is limited data linking proteomic and epigenomic studies to exposure itself (38).

Lastly, we acknowledge that the quantitative meta-analysis is limited by heterogeneity across included studies in study design, study sample sizes, and methodology for evaluating mesothelioma and health exposure. In particular, we understand that the pooled estimates and associated confidence intervals are biased by smaller studies with disproportional weighting and therefore, not representative of the true range of data. As such, we ensured that our forest plots depict the range of data across each of the included studies.

## Conclusion

In summary, we reviewed literature on non-occupational exposure setting in mesothelioma, and observed a discrepancy with sex, with more female cases having unknown or no history of exposure to asbestos. Future research should address changing modalities, environmental, and occupational sources of asbestos and other elongated fibers exposure in order to deploy public health initiatives that address such exposures in an upstream and timely fashion while reducing the downstream occurrence of this malignant disease.

## Data Availability

The original contributions presented in the study are included in the article/Supplementary material, further inquiries can be directed to the corresponding author.
