# Supplementary material for: Sex differences in asbestos exposure
Source: Front Public Health. 2025 May 22;13:1588415. doi: 10.3389/fpubh.2025.1588415 (PMC12137245; doi:10.3389/fpubh.2025.1588415)
Supplement: Supplementary file 1 [file Data_Sheet_1.docx]

Supplement Fig 1

Record identified through initial search: 1/1/2016 to 9/30/2024

(n=645)

Studies missing information of exposure type by sex (n=11)

Full-text articles excluded: reviews, case reports, commentaries (n=103)

Record excluded n=516

9 studies from previous review

+

Record screened (n=129)

Full-text articles assesses for eligibility (n=26)

Studies added to previous qualitative synthesis (n=15)

**24 studies included**
